# Comprehensive pan-carcinoma analysis of ITGB1 distortion and its potential clinical significance for cancer immunity

**DOI:** 10.1007/s12672-024-00901-9

**Published:** 2024-02-24

**Authors:** Yuchang Fei, Yulun Wu, Luting Chen, Huan Yu, Lei Pan

**Affiliations:** 1 Department of Integrated Chinese and Western Medicine, The First People’s Hospital of Jiashan, Jiashan Hospital Affiliated of Jiaxing University, Jiashan, Zhejiang China; 2 Center for Rehabilitation Medicine, Rehabilitation & Sports Medicine Research Institute of Zhejiang Province, Department of Rehabilitation Medicine, Zhejiang Provincial People’s Hospital, Affiliated People’s Hospital, Hangzhou Medical College, Hangzhou, Zhejiang China; 3 Department of Integrated Chinese and Western Medicine, The First People’s Hospital of Wenling, Wenling, Zhejiang China; 4 The Department of Traditional Chinese Medicine, The First Affiliated Hospital of Ningbo University, Ningbo, Zhejiang China; 5 Department of Oncology, The First Affiliated Hospital of Zhejiang Chinese Medical University, Hangzhou, Zhejiang China

**Keywords:** ITGB1, Pan-cancer, Bioinformatics, Immunity, Prognostic

## Abstract

The human protein-coding gene ITGB1 (Integrin 1), also known as CD29, has a length of 58048 base pairs. The Integrin family's most prevalent subunit, it participates in the transmission of numerous intracellular signaling pathways. A thorough examination of ITGB1's functions in human malignancies, however, is inadequate and many of their relationships to the onset and development of human cancers remain unknown. In this work, we examined ITGB1's role in 33 human cancers. Finally, a multi-platform analysis revealed that three of the 33 malignancies had significantly altered ITGB1 expression in tumor tissues in comparison to normal tissues. In addition, it was discovered through survival analysis that ITGB1 was a stand-alone prognostic factor in a number of cancers. ITGB1 expression was linked to immune cell infiltration in colon cancer, according to an investigation of immune infiltration in pan-cancer. In the gene co-expression research, ITGB1 showed a positive connection with the majority of the cell proliferation and EMT indicators, indicating that ITGB1 may have an essential function in controlling cancer metastasis and proliferation. Our pan-cancer analysis of ITGB1 gives evidence in favor of a further investigation into its oncogenic function in various cancer types.

## Introduction

Cancer, as a serious public unhealthiness worldwide, remains the second leading reason behind death worldwide and a major barrier to increasing expectancy [1]. The number of cancer cases worldwide is predicted to rise by 47 percent by 2040 to 28.4 million cases [2]. Due to its biological complexity and heterogeneity, and driven by environmental selective forces, cancer undergoes alterations in DNA methylation, histone, and transcriptome levels, which in turn trigger tumor progression [3], and existing biomarkers have limitations in predicting cancer prognosis. Moreover, the same gene may play different roles in different types of cancer. Pan-cancer analysis of cancer-associated genes to explore new biomarkers as prognostic and efficacy indicators for immunotherapy can better guide clinical work.

ITGB1, a subunit of the heterodimeric transmembrane receptor, is formed by binding to the ITGA subunit or the corresponding ligand [4] and is a member of the integrin-β (ITGB) superfamily, which is important in biological processes such as cell proliferation, carcinogenesis, and immune response [5]. It was found that overexpression of ITGB1 was significantly associated with advanced AJCC stage, histological grading, and poorer prognosis in pancreatic cancer patients [6]. In addition, a series of studies found that the integrin ITGB1, consisting of the β1 subunit, participates in promoting the progression of hepatocellular carcinoma, breast cancer, and pancreatic cancer, maintaining tumor cell viability and resistance to irradiation through the interplay of transduction of various intracellular communication pathways as key adhesion molecules [4, 7]. ITGB1 studies in cancer have so far been restricted to a particular cancer species; Therefore, ITGB1 must be subjected to a pan-cancer analysis in order to better understand its interactions with the underlying molecular mechanisms of action and the tumor immune microenvironment.

In the current study, utilizing data from various databases, we conducted the whole first pan-cancer assessment of ITGB1. The expression profile and prognostic significance of ITGB1 in a variety of cancers were investigated, and the findings demonstrated that ITGB1 can be used as a risk factor or prognostic factor for a number of malignancies, further emphasizing the possibility of ITGB1 as a prognostic biomarker for cancer and establishing a foundation for a thorough comprehension of ITGB1's function in tumor immunotherapy.

## Materials and methods

### Data collection and standardization criteria

**Fig. 1:**
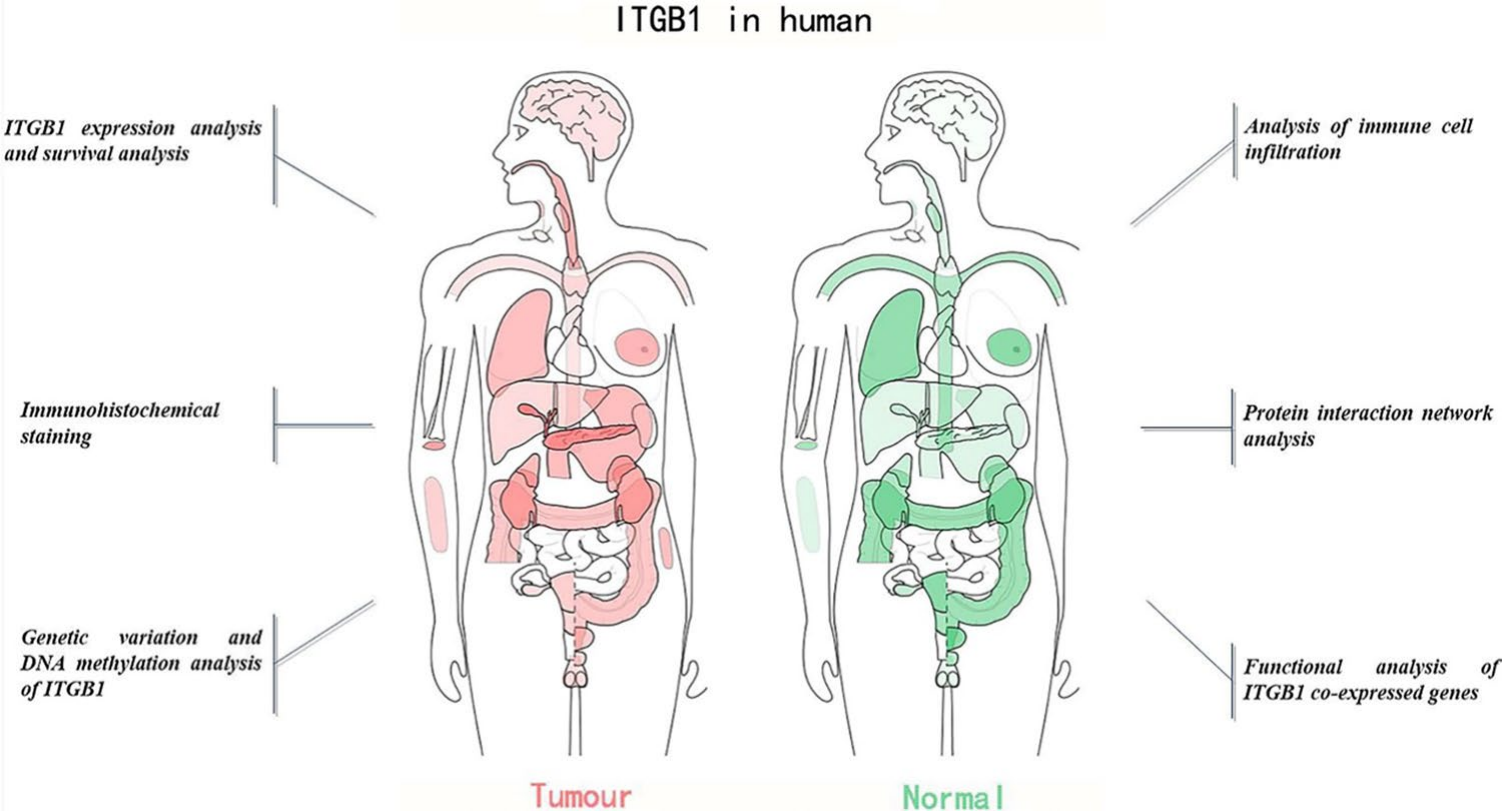
The workflow of this study and distribution of ITGB1 expression level from humans in GEPIA

The flow chart of this work is shown in Fig. 1. We used samples from The Cancer Genome Atlas (TCGA) and The Genotype-Tissue Expression (GTEx). Transcriptome RNA sequencing data of tumor and normal samples from the Project were analyzed for pan-cancer expression differences and transformed by log2(TPM + 1).

### ITGB1 expression analysis and survival analysis

Using TIMER 2.0 (http://timer.comp-genomics.org/), the expression of ITGB1 in tumor tissues and surrounding normal tissues was investigated in thirty-three cancer species [8]. In addition, the Survival Analysis module of GEPIA (http://gepia.cancer-pku.cn/index.html) was used to explore the relationship between ITGB1 and survival status in various cancers [9]. The median expression of ITGB1 was used as a cut-off for determining the group with high expression and the group with low expression.

### Validation of immunohistochemical results

We used the Human Protein Atlas database (https://www.proteinatlas.org/) to collect ITGB1 in different cancer types of immunohistochemical images. A total of 7 tumor tissues were included (Lung cancer, Head and neck cancer, Pancreatic cancer, Liver cancer, Urothelia cancer, and Ovarian cancer), Immunohistochemical images of Skin cancer and its symptomatic normal tissues were used to evaluate the difference in ITGB1 expression on a protein-based basis. We downloaded the ITGB1 staining information of cancer tissues and normal tissues in HPA.

### Genetic variation and DNA methylation analysis of ITGB1

Through the cBio Cancer Genomics portal (http://cbioportal.org), genetic alteration analysis was carried out [10]. Select the "PanCancer Studies" module, enter the "TCGA Pan-Cancer Atlas Studies" dataset, and select the "Cancer Types Summary" submenu to visualize the frequency of ITGB1 gene changes in pan-cancer. Used Gene Set Cancer Analysis (GSCA) (http://bioinfo.life.hust.edu.cn/GSCA/) [11] data Set assessment ITGB1 DNA methylation patterns, select "Mutation" module, selects all objects on the tumor type, Select the "CNV & Expression" submenu to visually analyze the relationship between copy number variation CNV and ITGB1 Expression.

### Analysis of immune cell infiltration

The following pan-cancer dataset was retrieved from the UCSC database: https://xenabrowser.net/. Using the R package "estimate", we generated the matrix, immunity, and estimation scores of each patient in each tumor from the TCGA Pan-cancer cohort. From this dataset, we further retrieved the ITGB1 gene expression data in each sample [12]. On the TISIDB platform, a correlation between ITGB1 and the quantity of TILs (tumor-infiltrating lymphocytes) was deduced [13]. In addition, TIDE, MCPCOUNTER, XCELL, and EPIC algorithms were used to estimate immunodeficiency. The Spearman's rank correlation test was used to determine P values. P < 0.05 was considered significant.

### Protein interaction network analysis

Using the STRING database [14] to identify ITGB1 potential binding partners with the following parameters: Meaning of Network Edges (Evidence), Active Interaction Sources (Experiments, Co-expression, Databases), the maximum number of participants to display (no more than 50 interactors), and the lowest required interaction score (Low confidence). This technique yielded a total of 50 ITGB1 interacting genes.

### Functional analysis of ITGB1 co-expressed genes

In order to further explore ITGB1 functions in cancer, in DAVID bioinformatics database (https://david.ncifcrf.gov/tools) [15, 16]. GO and KEGG analyses were performed on 200 genes most significantly related to ITGB1 expression, and the potential biological themes of 200 genes were obtained. The R package “ggplot2” was further used to draw the following biological functions and processes according to the enrichment analysis results: Molecular Function (MF), Cellular Component (CC), and Biological Process (BP).

### Statistical analysis

We transformed RNA-seq data obtained for 33 cancer types using log2(TPM + 1). The Kaplan–Meier method was used to assess the survival rate. To compute matrix, immunity, and estimated scores, utilize the "estimate" R package. The algorithms TIDE, MCPCOUNTER, XCELL, and EPIC were utilized to calculate immune infiltration. To determine the P-value, the Spearman rank correlation test was utilized. To create graphs depicting biological functions and processes, using the “ggplot2” R package. The tests mentioned above were all two-tailed, with a significance level of P < 0.05 being statistically significant.

## Results

### Expression and survival analysis of ITGB1 in normal and tumor tissues

**Fig. 2:**
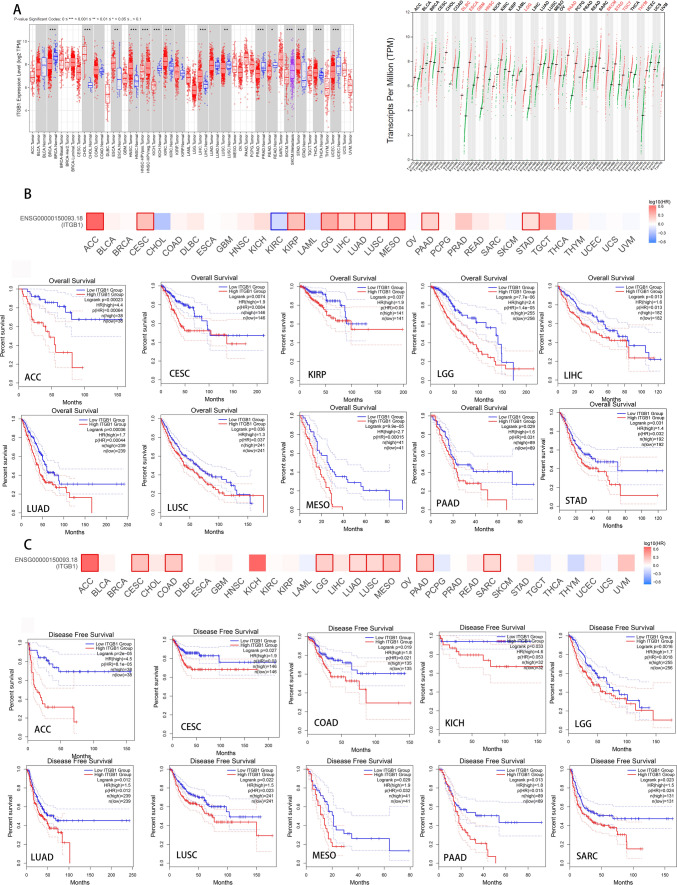
The analysis of differential level expression profile and prognostic value of ITGB1 in the TCGA cohort. **A** Analysis of ITGB1 expression in 33 cancers or cancer subtypes by online TIMER2.0 resource and GEPIA resource. *P < 0.05; **P < 0.01; ***P < 0.001. **B** Results of overall survival analysis of ITGB1 in pan-cancer. **C** Disease-free survival of ITGB1 in pan-cancer

To investigate the mRNA expression profile of ITGB1 in pan-carcinoma, we performed differential analyses using the "Gene DE" module of TIMER 2.0 and the "Single Gene Analysis" module of GEPIA. In TIMER 2.0, 7 cancer types had higher levels of ITGB1 expression relative to neighboring normal tissues (P < 0.05): CHOL (Colon Cancer), ESCA (Esophageal Cancer), HNSC (Head and Neck Cancer), KIRC (Kidney Clear Cell Cancer), LIHC (Liver Cancer), STAD (Stomach Cancer), THCA (Thyroid Cancer) (Fig. 2A). In addition, we found that ITGB1 was upregulated in 10 cancer types in GEPIA (Fig. 2B): cancer in DLBC (Large B-cell Lymphoma), ESCA (Esophageal Cancer), GBM (Glioblastoma), HNSC (Head and Neck Cancer), and LGG (Lower Grade Glioma), PAAD (Pancreatic Cancer), SKCM (Melanoma), STAD (Stomach Cancer), TGCT (Testicular Cancer), THYM (Thymoma). Finally, the intersection of the two up-regulated cancer types revealed that ITGB1 expression was up-regulated in ESCA, STAD, and HNSC.

**Fig. 3:**
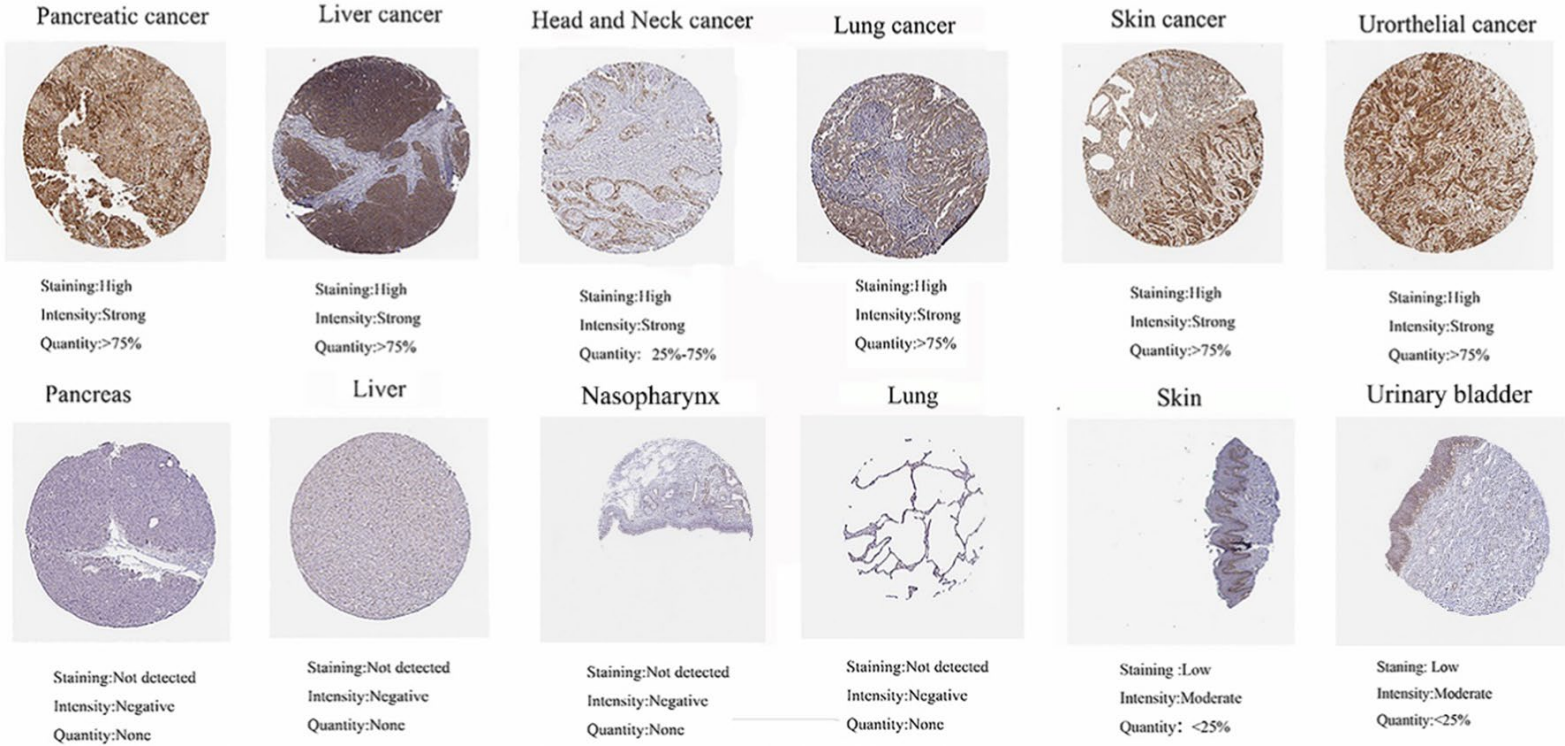
Protein expression of ITGB1 based on immunohistochemical staining. Results of IHC differences between 6 types of cancer and non-tumor tissues

We looked into ITGB1's prognostic importance in 33 distinct cancer types in further detail. According to Fig. 2B, in the following cancer types: ACC, CESC, KIRP, LGG, LIHC, LUAD, LUSC, MESO, PAAD, and STAD (P < 0.05), poor overall survival was linked to higher ITGB1 expression (OS) (Fig. 2B).In addition, disease-free survival (DFS) analysis suggested that ITGB1 upregulation was associated with ACC, CESC, COAD, KICH, LGG, and LUAD, USC, MESO, PAAD, and SARC were significantly associated with poor prognosis (P < 0.05) (Fig. 2C). These results suggested that in ACC, CESC, MESO, LUSC, and PAAD, ITGB1 is a unique prognostic predictor for DFS and OS. We also used IHC data from the "Human Protein Atlas" database to look at the ITGB1 protein levels in various cancer types. The findings demonstrated that compared to similar normal tissues, cancerous tissues of the lung, head and neck, pancreas, liver, skin, and urothelium displayed increased ITGB1 expression (Fig. 3). The IHC results that showed ITGB1 protein levels agreed with our analysis.

### Analysis of genetic and epigenetic variation

**Fig. 4:**
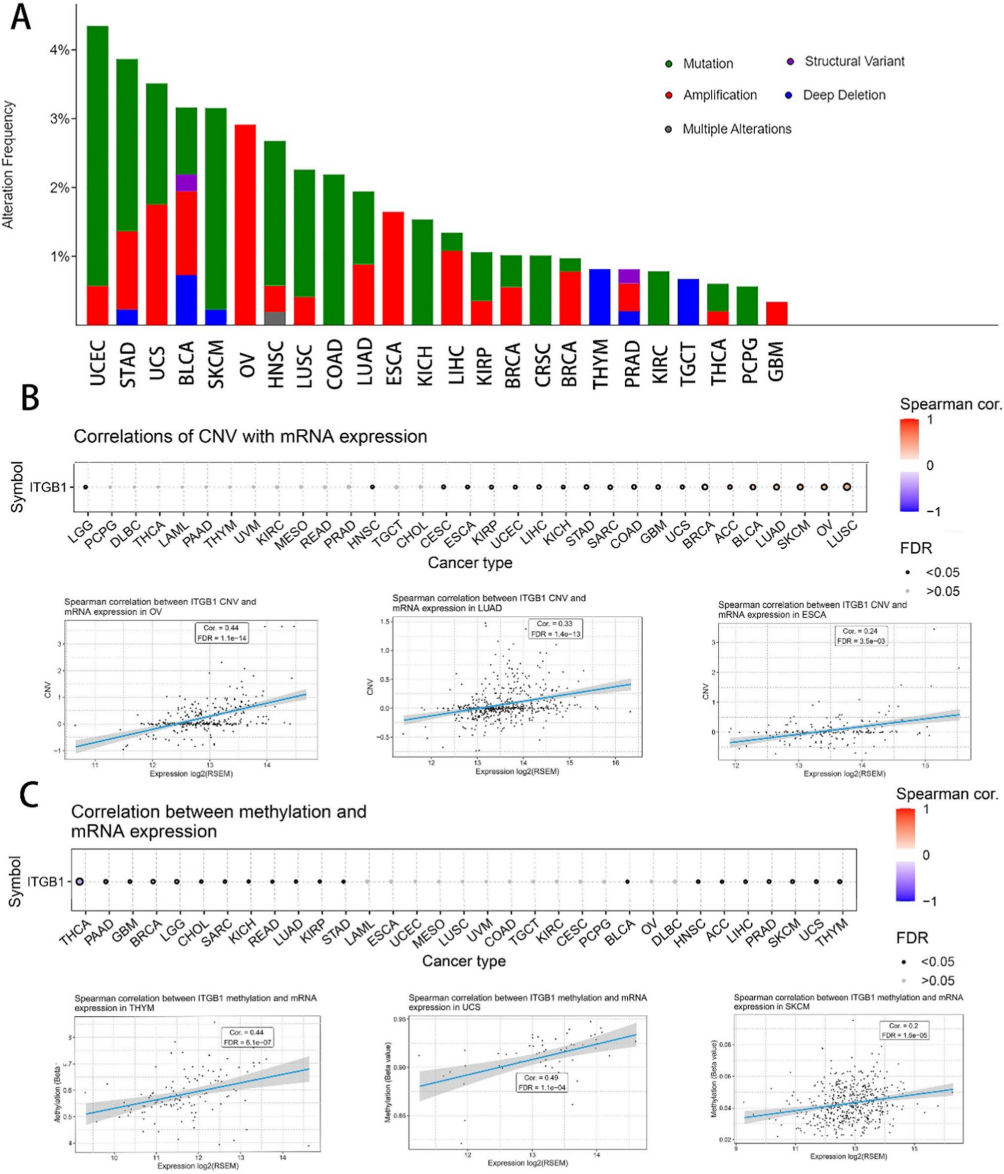
Genetic and epigenetic changes in ITGB1 in pan-cancer. **A** ITGB1 mutational profiling of tumor groups in the TCGA cohort was performed using the cBioPortal online tool. **B** The relationship between copy number variation (CNV) and ITGB1 expression level was analyzed by GSCA method. **C** Correlation analysis between ITGB1 expression level and DNA methylation in pan-cancer

We investigated ITGB1 gene alterations in the TCGA cancer profiling study using cBioPortal. The overall frequency of genetic alterations in ITGB1 was found to be relatively low in pan-cancer studies (Fig. 4A). The highest Mutation frequency of ITGB1 in UCEC was 4.35%, mainly of the "Mutation" type. However, no gene changes were found in LAML, ACC, CHOL, DLBC, MESO, PAAD, SARC, and UVM. We concentrated on ITGB1 DNA fragments with copy number amplification due to the overexpression of ITGB1 in diverse malignancies. OV (2.91%), UCS (1.75%), and ESCA (1.65%) had greater amplification frequencies. Additionally, we looked into the potential connection between ITGB1 expression and copy number amplification in 33 malignancies. Figure 4B illustrates the positive correlation between 20 tumors that we found (FDR < 0.05).

One of the essential epigenetic pathways for the development of cancer is abnormal DNA methylation [17]. We used the GSCA method to assess the DNA methylation patterns of ITGB1. In 20 tumors, it was discovered that DNA methylation was adversely linked with the level of ITGB1 expression (Fig. 4C) (FDR < 0.05). Combined with the top knowledge analysis results, we have a tendency to conclude that desoxyribonucleic acid copy variety amplification and methylation area unit 2 potential causes of ITGB1 upregulation in cancer.

### Co-expression analysis of the ITGB1 gene

**Fig. 5:**
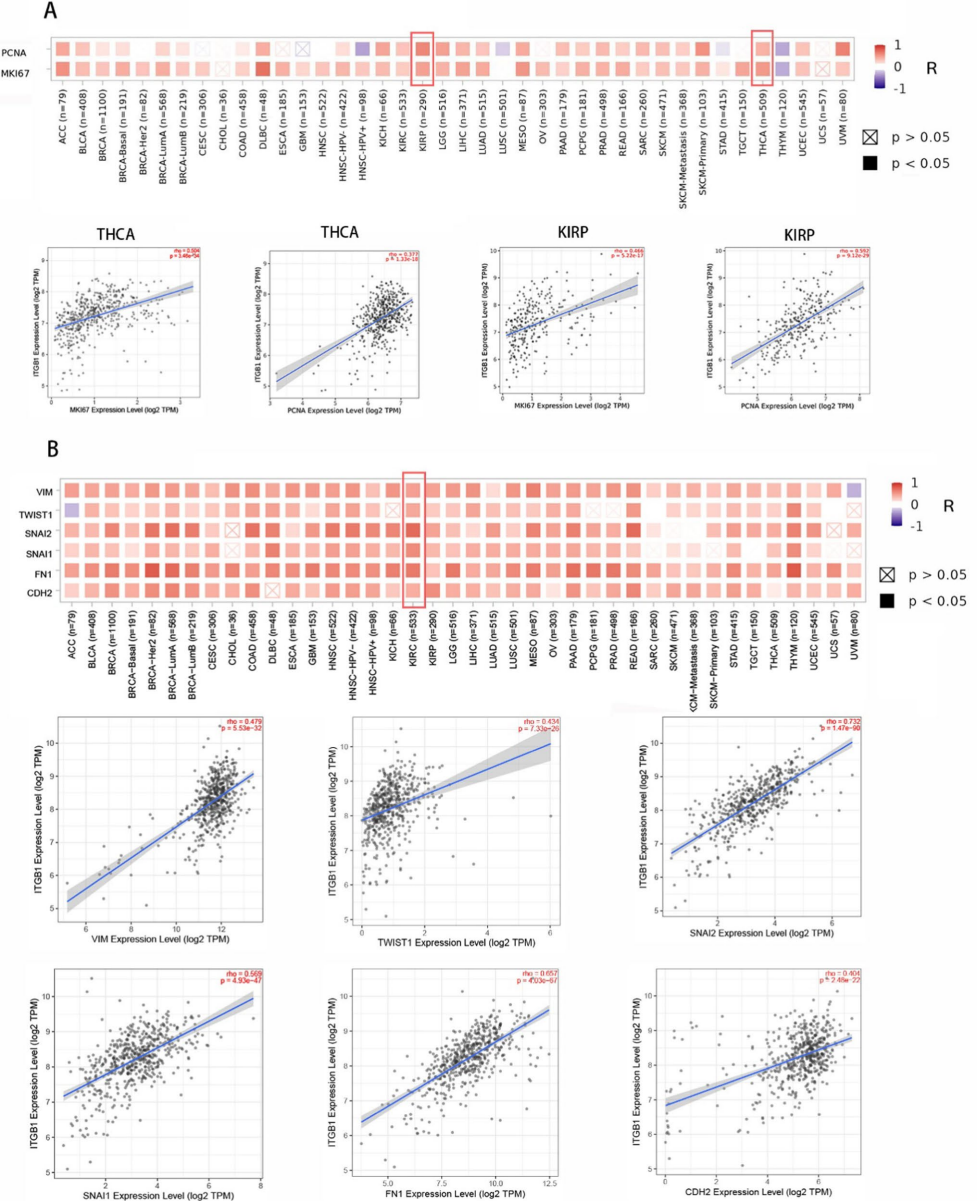
Gene co-expression analysis of ITGB1 with markers of epithelial-mesenchymal transition and cell proliferation. **A** Relationship between ITGB1 expression levels and proliferation markers (MKI67 and PCNA) in pan-carcinomas. **B** Correlation analysis between ITGB1 expression and EMT markers (Vimentin, TWIST1, Snail1, Snail2, Fibronectin 1, CDH2)

To further investigate the potential role of ITGB1 in multiple cancers. We performed the analysis through gene co-expression networks. It has been confirmed that tumor proliferation-related markers MKI67 and PCNA (proliferating cell nuclear antigen) are closely related to the differentiation, invasion, and metastasis of many tumors. We focused on the potential link between ITGB1 and MKI67 and PCNA in this study. NUTF2 expression was found to be highly correlated with MKI67 and PCNA in 27 tumor types including ACC, BLCA, and BRCA (P < 0.05). (Fig. 5A). Epithelial-mesenchymal transition (EMT) is the key to tumor progression, which gives cells the ability to metastasize and invade. Based on this, we further performed ITGB1 and EMT-related markers: The correlation between Vimentin (VIM), TWIST1, Snail1 (SNAI1), Snail2 (SNAI2), Fibronectin 1 (FN1), and N-cadherin (CDH2) was analyzed. The analysis's findings showed that ITGB1 was completely related to the expression of EMT-related genes (Fig. 5B). These findings suggest that ITGB1 may be involved in tumor proliferation and invasion.

### Matrix and immune infiltration analysis of ITGB1

The stroma is currently believed to be the connective tissue of biological tissues and to be crucial to both healthy wound healing and malignancy. However, in the case of cancer, uncontrolled cell proliferation and invasion, metastasis, and therapeutic tissue resistance can all come from it [18–20].

**Fig. 6:**
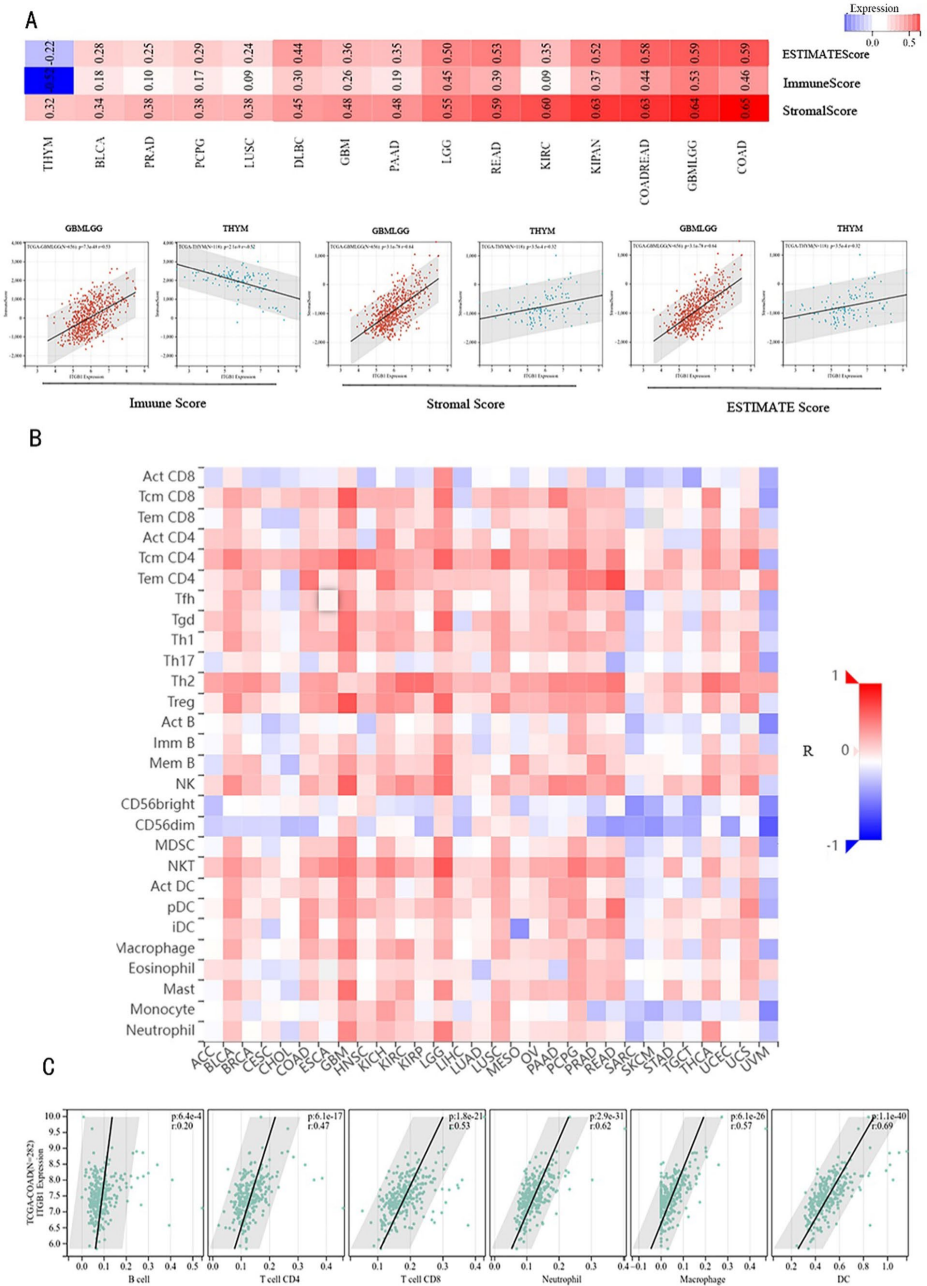
The correlation between ITGB1 expression and abundance of tumor-infiltrating lymphocytes (TIL) was analyzed. **A** Explore potential associations between infiltrating stroma and immune cells and ITGN1 expression levels using the ESTIMATE algorithm. **B** correlation analysis between abundance of tumor-infiltrating lymphocytes and ITGB1 expression in pan-cancer. **C** Correlation analysis of ITGB1 expression with B cells, CD4 T cells, CD8 T cells, macrophages, neutrophils and dendritic cells in COAD

In addition, the biology of solid tumors is largely determined by the interaction between cancer cells and their surrounding microenvironment, mediated by various combinations of immune cells, which participate in the cross-talk between the immune microenvironment and tumor cells [20]. In this work, the potential association between immune cells and invading matrix, as well as the level of ITGB1 expression, were investigated using the ESTIMATE formula. The findings of the analysis revealed that in 15 cancer species, ITGB1 expression was substantially linked with an immune score, a matrix score, and an ESTIMATE score (Fig. 6A). Notably, ITGB1 expression level was completely related to with associated immune score, stromal score, and ESTIMATE score in READ (Fig. 6B).

Interestingly, ITGB1 expression was inversely correlated with immune/matrix/ESTIMATE score in SARC (Fig. 6B, C). This information demonstrates a possible role for ITGB1 in controlling the neoplasm microenvironment (TME).

### Relationship between ITGB1 and immune cell markers

**Fig. 7:**
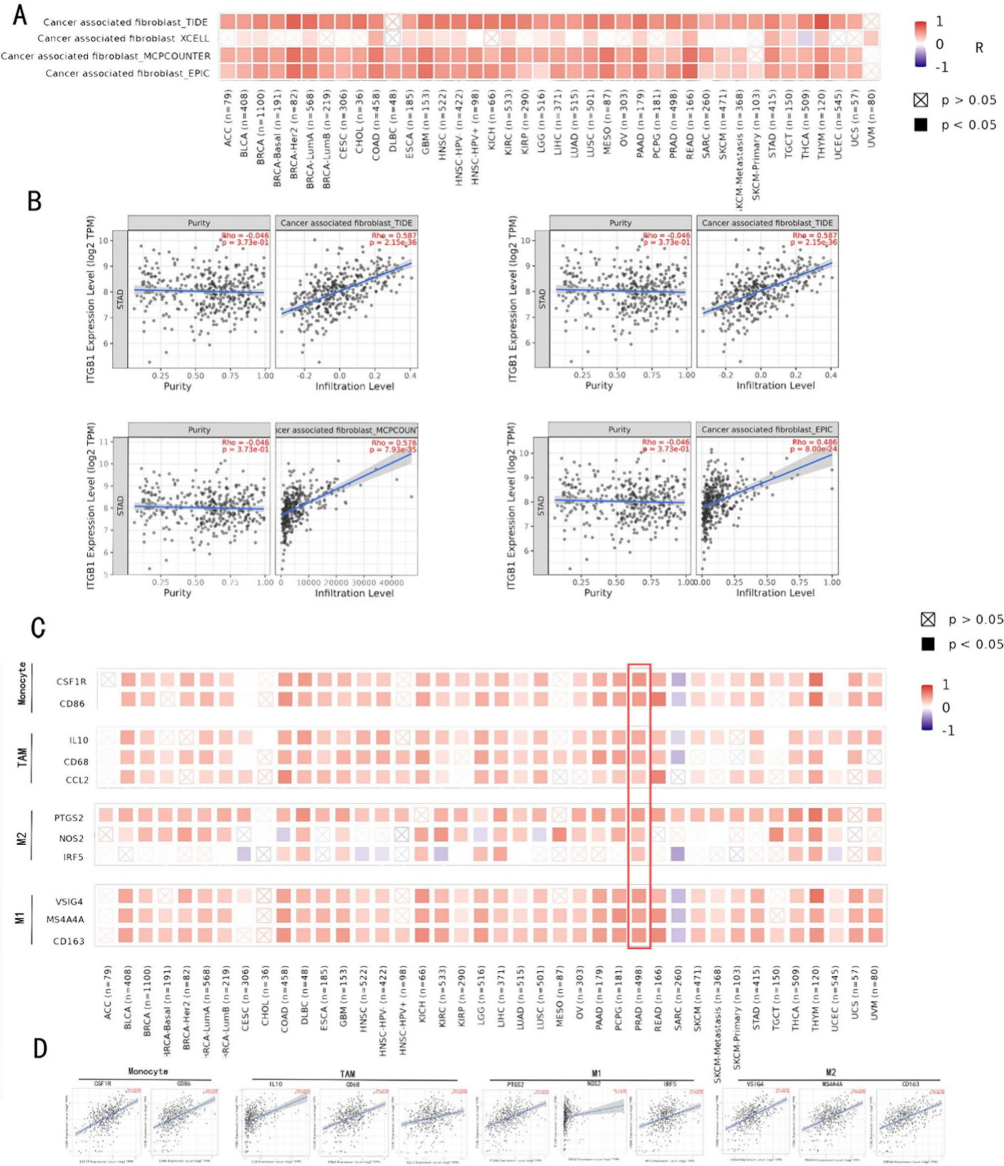
Correlation analysis between immune cells and ITGB1. **A** The potential association between ITGB1 expression levels and cancer-associated fibroblast (CAF) infiltration was explored by four algorithms. **B** The correlation between ITGB1 expression and infiltrating CAF in STAD was analyzed by EPIC or TIDE algorithm. **C** Correlation analysis between ITGB1 expression and immunolabeling-related genes in all cancers in the TCGA cohort. **D** Correlation analysis between ITGB1 expression and thymus monocytes, TAMs, M1 macrophages, and M2 macrophages

A very heterogeneous interstitial cell type called cancer-associated fibroblasts (CAFs) is linked to the development, growth, and metastasis of tumors [21]. They encourage the development of cancer cells by secreting growth factors, inflammatory ligands, and extracellular matrix proteins, which leads to treatment resistance and immunological rejection [22]. We used four algorithms (EPIC, MCPCOUNTER, XCELL, TIDE) to investigate the correlation between ITGB1 and cancer-associated fibroblasts (Fig. 7A, B). The results showed that CAFs and ITGB1 expression were positively correlated in 18 cancer types (P < 0.05 in all four algorithms) (Fig. 7A).

Another important element contributing to the growth of tumors has recently been recognized as the interaction between CAFs and the tumor immune microenvironment (TIME). TIME consists mainly of different populations of immune cells in the tumor islets. Various cytokines released by CAFs interact with tumor-infiltrating immune cells and other immunological components in ITIME to generate immunosuppressive TME, which allows cancer cells to avoid immune system surveillance [23]. The link between ITGB1 and immune-related genetic markers was further examined in this study. Tumor-associated macrophages (TAM), M1 and M2 macrophages, and monocytes made up the majority of the immune cells. The results demonstrated a substantial correlation between the expression of ITGB1 in THYM, THCA, LIHC, PRAD, LGG, KIRC, COAD, and DLBC with the expression of TAM, M1 macrophages, M2 macrophages, and monocytes (P < 0.05). (Fig. 7C, D). Among them, ITGB1 in THYM, THCA, LIHC, PRAD, and DLBC was positively correlated with monocytes, TAMs ( MI macrophages, and M2 macrophages (all P < 0.05) (Fig. 7C). Notably, ITGB1 of CARC was negatively correlated with M2 macrophages and monocytes (P < 0.05). The role of ITGB1 in the tumor microenvironment (TME) and its interaction with ITEM remains to be further confirmed.

### Enrichment analysis of ITGB1 co-expressed genes

**Fig. 8:**
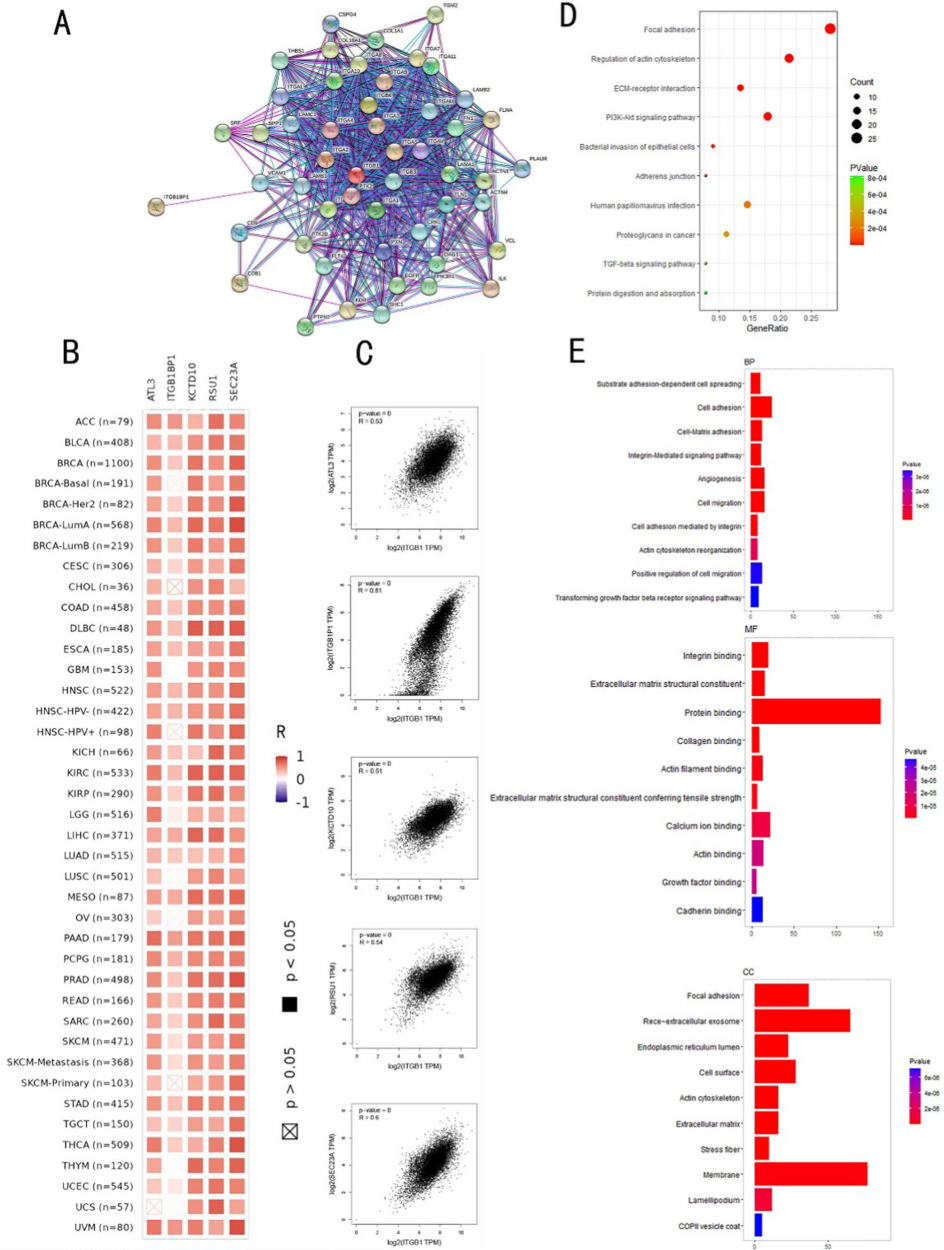
Gene co-expression enrichment analysis of ITGB1. **A** Analysis of the protein–protein interaction network (PPI) by STRING platform. **B** Heatmap of ITGB1 and the top 5 related genes in pan-cancer. **C** Scatter plot of the association between the top 5 genes and ITGB1 in pan-cancer. **D** KEGG pathway analysis was performed on the top 200 genes related to ITGB1 expression, and the top 10 signaling pathways were shown in the bar graph. **E** Gene ontology analysis of the top 200 genes, including Biological Process (BP), Molecular Function (MF), and Cellular Component (CC)

We used the STRING online resource to conduct protein–protein interaction network analysis (PPI) in order to further investigate the probable molecular mechanism of ITGB1 in the emergence of cancer. A total of 50 ITGB1-binding proteins with experimental evidence were obtained (Fig. 8A). We obtained the top 200 genes most related to ITGB1 by using the "Similar Genes Detection" module of GEPIA 2, and selected the top 5 genes for correlation analysis (Fig. 8B, C): ITGB1P1(R = 0.81), SEC23A(R = 0.6), RSU1 (R =0.54), ATL3(R = 0.53), KCTD10(R = 0.51). The expression of ITGB1 was positively linked with the top five genes in the majority of genes (P < 0.05) [24–26]. It is concerning because recent research has revealed a link between high levels of SEC23A and RSU1 expression and poor prognosis in a number of malignancies, indicating that these proteins may have pro-cancer actions. High expression of KCTD10 is associated with a good prognosis of cancer [27].

The first 200 genes' functional enrichment analysis, however, uncovered numerous cancer-related pathways, including the P13K-Akt signaling network, Proteoglycans in cancer, and Adherens junction human (Fig. 8D). We conjointly specialize in factor ontologies associated with biological processes, cellular composition, and molecular functions. Studies have found that "Cell adhesion" and "Protein binding" may be involved in the role of ITGB1 in cancer pathogenesis (Fig. 8E). These results shed light on the molecular mechanisms through which ITGB1 may be involved in tumorigenesis.

## Discussion

ITGB1, also known as Integrin β1 (also known as CD29), is a human protein-coding gene. The heterotomic transmembrane receptor's ITGB1 subunit is created when the ITGA subunit or the appropriate ligand binds to it. The most prevalent integrin subunit is called ITGB1. Currently, it is known that integrin 1 binds to various subunits to create 11 distinct integrins. These integrins are widely dispersed throughout the body and are essential for mediating extracellular matrix interactions [28]. Numerous studies have demonstrated that ITGB1, an integrin that belongs to the 1 subgroup, regulates cell–matrix contact and is essential for sustaining tumor stemness, proliferation, dissemination, metastasis, radiotherapy, chemotherapy, and medication resistance [7, 29]. According to Li et al., higher expression of ITGB1 was a separate risk factor for osteosarcoma and was linked to patients' poor prognoses [30]. The research by Yin et al. revealed that ITGB1 can control the triple-negative breast cancer (TNBC) cells' invasion, migration, and treatment sensitivity and may be a predictive biomarker for the survival of TNBC patients. In the investigation of HCC, it was discovered that ITGB1 knockdown dramatically reduced HCC invasion and migration, indicating that ITGB1 is involved in the development of HCC [31]. A number of research have also revealed that ITGB1 aids in the expansion and invasion of gastric cancer, non-small cell lung cancer, and pancreatic cancer [7, 32–34].

This study looked into the expression of ITGB1 in 33 cancers from the TCGA database. In 7 cancers using the TIMER 2.0 platform and 10 cancers utilizing GEPIA, ITGB1 expression was considerably increased in comparison to normal surrounding tissues. Finally, a comparison of the two data platforms revealed that ITGB1 was highly elevated in ESCA, STAD, and HNSC. The expression of ITGB1 was considerably higher in gastric adenocarcinoma than in normal tissues, and it was also higher in the tumor tissues of individuals with advanced cancer (P < 0.05) [35]. With a positive association with immunosuppressive variables and a negative correlation to immunoreactive factors [36], ITGB1 has also been discovered as a predictive biomarker linked to immunosuppression in gastric cancer [37]. In esophageal cancer, ITGB1 has been found to be associated with lymph node metastasis and poor prognosis, which can be used as an independent prognostic factor and confer resistance to chemotherapy in esophageal cancer [38]. In addition, it has been previously found that overexpression of ITGB1 may be associated with chemoresistance in esophageal cancer. Targeting ITGB1, especially in patients treated with docetaxel, may enhance the efficacy of chemotherapy in patients with esophageal cancer [39]. Notably, we also discovered that ITGB1 was significantly expressed differently in HNSC-HPV + and HNSC-HPV -. HNSC is typically linked to smoking, alcoholism, or both and is mostly obtained from the mucosal epithelium of the oral cavity, pharynx, and larynx. HPV infection is a clear risk factor for pharyngeal cancer [40]. A multicenter retrospective study found that WHO type I nasopharyngeal carcinoma was associated with HPV infection [41]. Hpv-positive NPC tends to have a poor prognosis [42]. It has been shown that the conserved C-terminal sequence of the E6 protein of high-risk cutaneous β-HPV promotes cell migration by altering β1-integrin localization and signaling, which may be involved in contributing to the pathogenicity of these β-HPV types [43].

Cancer genomics has recently offered fresh perspectives on the genetic changes and signaling mechanisms in cancer [44]. Studies have shown that aberrant DNA methylation is a hallmark of cancer. Since the incidence of thyroid cancer is increasing year by year, it is necessary to explore DNA methylation in thyroid cancer. Our analysis found that the expression level of ITGB1 in thyroid tumors was considerably correlated with polymer methylation, suggesting that ITGB1 could have a very important role in the progression and prognosis of thyroid cancer. To explore the role of ITGB1 in THCA, we have a tendency to additionally analyzed the link between ITGB1 and proliferation markers (PCNA, Ki67) and EMT markers (VIM, TWIST1, SNAI1, SNAI2, FN1, and CDH2). We discovered a positive correlation between the expression of ITGB1 and KI67 and PCNA. Additionally, VIM, TWIST1, SNAI1, FN1, and CDH2 had favorable correlations with the expression of ITGB1. This suggests that ITGB1 may be a potential marker for thyroid cancer proliferation and metastasis. The extracellular matrix (ECM), blood arteries, fibroblasts, lymphocytes, bone marrow-derived inflammatory cells, signaling chemicals, and surrounding immune cells make up the tumor microenvironment (TME). The tumor microenvironment plays a crucial role in the growth and malignancy of solid tumors by helping to maintain tumor stemness and directly promoting tumor angiogenesis, invasion, metastasis, and chronic inflammation [32, 45]. Immune cells that have invaded tumors are crucial for either encouraging or preventing tumor growth [46]. Numerous studies have shown that CD8 + T lymphocytes and Tregs show higher immune infiltration in tumor cells [47]. As a component of the tumor microenvironment, tumor-infiltrating B lymphocytes (TIB) exist in all stages of cancer, playing both pro-tumor and anti-tumor roles. Previous studies have demonstrated a strong correlation between a high immune/stromal/ESTIMATE score and a poor prognosis [48] and the advancement of tumor grade in LGG, which suggests that ITGB1 also contributes to the progression of cancer by boosting stromal and immune cell infiltration in LGG. Low immune/matrix/ESTIMATE scores, in contrast, were linked to a poor prognosis and an advanced tumor stage in SARC [49]. It has been verified that tumor-associated macrophages have a determining role in the induction of immune tolerance of different cancers [50]. Recently, Batchu et al. demonstrated that tumor-associated macrophages inhibit immune response through interaction with ITGB1 [51]. This study discovered that ITGB1 expression was adversely correlated with immune/stromal/ESTIMATE scores in SARC, indicating that ITGB1 was negatively correlated with immunological tolerance, potentially through tumor-associated macrophage contact with T cells. Immune infiltration mediated by ITGB1 may be one of the causes of carcinogenesis.

Cancer-associated fibroblasts (CAFs) constitute very heterogeneous stromal cells and are an important part of the microenvironment of solid tumors. The complex interactions of CAFs with other cell types in the TME are actively involved in cancer progression. Growth factors, inflammatory ligands, and extracellular matrix proteins secreted by CAF encourage the growth of cancer cells, the development of treatment resistance, and immunological rejection [22]. Recent studies have shown that CAFs may also inhibit tumor progression under certain circumstances. Recent studies have highlighted the emerging role of CAFs in immune regulation, which shape the tumor immune microenvironment (TIME) and influence the response to cancer immunotherapies [52]. A unique CAF population, termed antigen-presenting CAF (apCAF), has a role in modulating tumor immunity. During the progression of pancreatic cancer, mesothelial cells downregulate mesothelial characteristics and acquire fibroblast characteristics to form apCAF, which directly connects and induces naive CD4 + T cells to transform into regulatory T cells (Tregs) in an antigen-specific manner, thus playing a role of immune infiltration [53]. Exosome-mir-522 in CAF inhibits ferroptosis and promotes the acquisition of chemical resistance in gastric cancer by targeting ALOX15 and blocking lipid ROS accumulation [54]. A breast tumor subtype known as triple-negative breast cancer (TNBC) is extremely aggressive and has a dismal prognosis. Previous studies have found that LRRC15 in CAFs promotes the migration and invasion of TNBC cells through the Wnt/β-catenin signaling pathway [55]. Existing research shows that the tumor microenvironment of different components (including cancer-associated fibroblasts) associated with cancer through different mechanisms (including immune ignorance) enhanced radiation resistance [50]. Recent research has also further explore the mitochondrial metabolism in cancer progression [56] and the key role in the immune escape [57]. Research has proved the ITGB1 can participate in the mitochondria [58]. In this study, we found that ITGB1 CAFs are expressed in the 18 types of cancer are related (in four kinds of algorithms are all p < 0.05), suggesting that ITGB1 is likely to be involved in the conversion of CAFs and activation, and promote the development of cancer. As mentioned in the introduction, this correlation may be the root cause of the poor response to radiation.

In-depth analysis of the ITGB1 expression profile in human cancers, copy number variation, and DNA methylation in pan-cancer, as well as further investigation of the relationship between ITGB1 expression level and biological features of tumor cells and tumor immunity, were all provided by this study. Therefore, our study provides an accurate and detailed reference for a comprehensive understanding of the role ITGB1 plays in tumorigenesis, progression, and prognosis. We found that ITGB1 is also a helpful biomarker for determining the identification and prognosis of human cancers, however additional in vivo and in vitro validation experiments square measure still required to know the mechanisms of ITGB1 action in several cancer sorts at the cellular and molecular levels.

## Data Availability

The orignal data supporting this research are from online repositories. It was used to analyze RNA-sequencing expression data from 9736 tumors and 8587 normal samples from the TCGA and GTEx projects with the use of standard processing algorithms. The relevant positions in the text of these prior data set sources are cited as references.
